# Ultra-processed food for infants and toddlers; dynamics of supply and demand

**DOI:** 10.2471/BLT.22.289448

**Published:** 2023-04-06

**Authors:** Elizabeth K Dunford, Barry M Popkin

**Affiliations:** a Department of Nutrition, Gillings Global School of Public Health, The University of North Carolina at Chapel Hill, Chapel Hill, United States of America (USA).; b Carolina Population Center, The University of North Carolina at Chapel Hill, Chapel Hill, USA.

Historically, much of the discussion surrounding infant feeding focused on promoting breastfeeding the first six months (at least) of an infant’s life, and avoiding breastmilk substitutes. In 1981, the World Health Organization (WHO) developed the International Code of Marketing of Breast-Milk Substitutes to restrict the marketing of breastmilk substitutes.^1^ However, other beverages and toddler foods do not have the same marketing restrictions under the code, and therefore formula companies have broadened into other sections of infant and toddler feeding such as ultra-processed baby foods. The definition of infant formula is milk formula directed at children aged 0–12 months; it excludes growing-up formula as milk formula directed at children aged 12–36 months, and baby food as all packaged foods and/or beverages marketed to infants and toddlers aged 6–36 months.

The world is experiencing a continued global transition towards increasing consumption of infant formula, growing-up formula and processed baby foods:^2^ the global infant and toddler food market is projected to reach 120 billion United States dollars (US$) by 2030.^3^

This increased consumption represents a critical change in infant and toddler food habits as historically, food habits were centred around minimally processed food. The shift towards feeding infants and toddlers ultra-processed foods brings health risks such as increases in abdominal obesity and worse future health outcomes, and may impact future food preferences.^4^^,^^5^

A key shift in the infant and toddler food sector is in the amount of sugar added to these foods. Excess sugar consumption is a primary cause of obesity and related diseases, including diabetes, heart disease and some cancers.^6^ WHO guidelines recommend that less than 10% of total calories come from added sugar, preferably less than  5%,^6^ to reduce the risk of non-communicable diseases and prevent and control unhealthy weight gain and dental caries. However, these guidelines were developed for children older than 2 years and adults.

Here we use data from Euromonitor International^7^ to assess sales trends of milk formula, growing-up formula and baby foods during the past decade as well as the level of sugar sold in the infant and toddler feeding sector. 

## Trends in milk formula sales

**Fig. 1:**
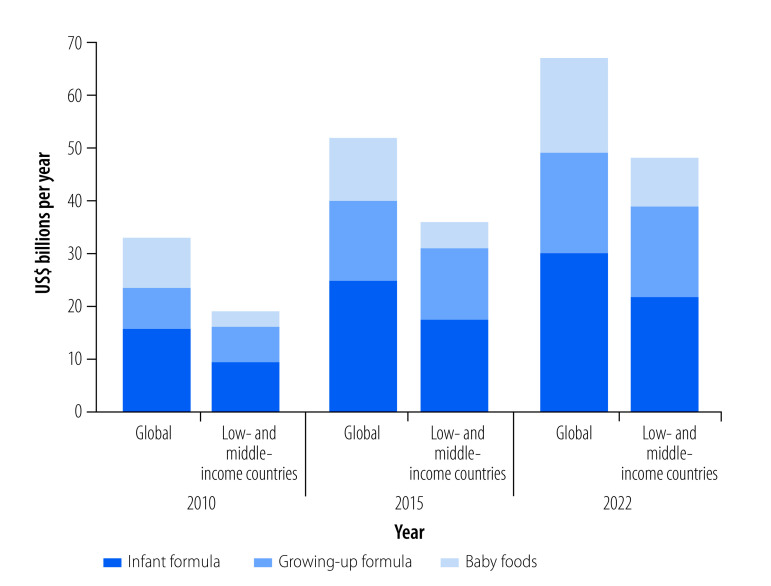
Total global sales from the infant and toddler feeding sector, 2010–2022

Between 2010 and 2022, global sales in the infant and toddler feeding sector overall grew from US$ 33.2 billion to US$ 67.9 billion (Fig. 1). Infant formula has historically represented the largest proportion of product sales globally. However, the proportion of sales from growing-up formula is increasing and has grown from 23% of the sector in 2010 (US$ 8.0 billion) to 28% in 2022 (US$ 19.3 billion; Fig. 1). Over 90% (US$ 17.5 billion/US $19.3 billion) of growing-up formula sales in 2022 took place in low- and middle-income countries. Even higher-income countries are seeing a rise in growing-up formula (from US$ 1.2 billion in 2010 to US $1.7 billion in 2022), with a concurrent plateauing in the proportion of sales deriving from infant formula, from 46% (US$ 6.5 billion/US$ 14.1 billion) in 2010 to 46% (US$ 8.7 billion/US$ 19.1 billion) in 2022. The increase in growing-up formula sales raises serious concern for global child health, particularly in some Asian countries where sales dominate, and calls into question the efficacy of current regulations designed to promote optimal infant feeding practices. 

Data source: Euromonitor International, 2022.^7^

US$: United States dollars.

## Baby food market

The global baby food market has grown from US$ 9.6 billion in 2010 to US$ 17.9 billion in 2022 (Fig. 1). Emerging economies offer opportunities for baby food manufacturers and now represent half of all global baby food sales. Driving factors in this increase include change in lifestyle, higher presence of women in the workforce and increase in birth rates.^2^ Furthermore, the nutrients and ingredients used in baby foods are changing. Increases in added sugar, increased use of additives and a wider range of ingredients are being used globally in processed foods, and baby foods are no exception. The consumption of commercially prepared baby foods in many cases may exceed consumption of homemade foods for infants and toddlers. Concerns exist about the nutritional composition, sweet taste and long-term health effects of these products. 

## High-sugar foods

**Fig. 2:**
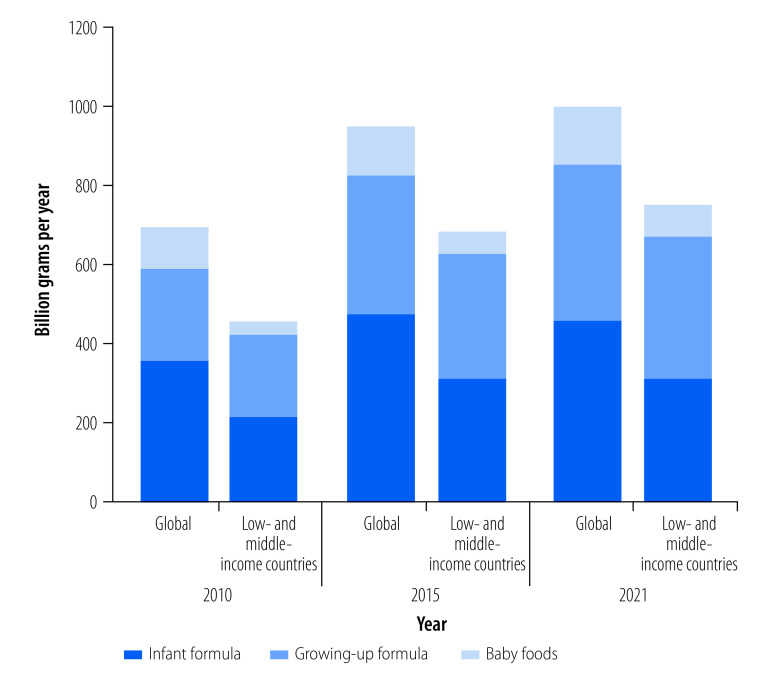
Sugar sales from the infant and toddler feeding sector, 2010–2021

An increase in sales of sugar through the infant and toddler food sector took place, from 697 billion grams in 2010 to 1009 billion grams in 2021 (Fig. 2). Research conducted in the United States of America has shown that most baby foods contain added sugars and/or are high in sodium.^8^ One in four baby foods in South Africa contain added sugars,^9^ research from Australia showed that infant and toddler snack and meal products launched after 2014 had a higher median sugar content compared to 1996^10^ and research from India in 2021 found almost half of baby foods were high in sugar (that is, more than 20% of calories deriving from sugar).^11^ Using data from Mintel’s Global New Products Database in India,^12^ it is clear that growing-up milks with added sugars are higher in mean total sugar content than infant formula and baby foods, and much higher than the sugar content in cow’s milk.

Data source: Euromonitor International, 2022.^7^

## Ultra-processed foods

The increase in availability and variety of baby foods comes at a time when we are learning that the consumption of ultra-processed foods containing multiple ingredients and additives poses health risks. Taste preferences and dietary habits formed during early childhood can persist into adulthood. The diets of infants and toddlers in the United States and across high-income countries are dominated by ultra-processed foods.^13^ Longitudinal studies have clearly demonstrated a positive association between the consumption of ultra-processed foods and obesity in young children.^14^ Little doubt exists that children globally are being increasingly exposed to these foods, with substantial expansion in the types and quantities sold. Although intakes of ultra-processed foods are highest in high-income countries, they are growing rapidly in low- and middle-income countries. These ultra-processed foods represent a threat to infants, toddlers and pre-schoolers’ health.

## Discussion

The data presented here raise major questions about the role infant and toddler food companies play in rising global obesity levels and the double burden of malnutrition in low- and middle-income countries. Today every country has more than one fourth of their adult population overweight and not a single country has seen a significant decrease in adult overweight and obesity in recent decades.^15^ The presence of added sugar is one defining element of ultra-processed foods, and most products containing added sugar would be considered ultra-processed. These large formula companies are hence further contributing to the global sweetening of human diets via their inclusion of added sugar in products directed towards infants and toddlers.

Given the increasing evidence base demonstrating the adverse long-term health effects of consumption of ultra-processed foods and added sugars, global concerns now exist for infant and child health. By definition, milk formulas themselves are ultra-processed foods, typically consisting of powdered milk proteins, vegetable oils, lactose and other added sugars, micronutrients and additives. The five largest global formula companies are Nestlé, Danone, Abbott, RFC and RBMJ with four out of these five companies present in more than 100 countries.^16^ Our data clearly show that these companies are focusing their growth, particularly through growing-up formula, in low- and middle-income countries. Decades ago, major legal trials took place in Switzerland against Nestlé, the global leader in infant formula. These trials led to the development of the WHO International Code of Marketing of Breast-Milk Substitutes. However, despite this code and the conclusive science showing the harms of increased consumption of ultra-processed foods, the data presented here demonstrate that the infant and toddler food industry has managed to sustain and even increase sales both globally and in low- and middle-income countries. 

The diets of infants and young children worldwide are undoubtedly becoming increasingly highly processed, a trend mirrored by increased global consumption of ultra-processed foods. Policy-makers need to ensure these products and the companies who manufacture them are monitored closely, and marketing of these products is more tightly regulated. Future research should investigate options for regulatory measures that could address products that currently lie outside the scope of the code. Doing so will be of critical importance in low- and middle-income countries, where socioeconomic factors and the broader food environment put vulnerable populations at risk.
